# Outer Membrane Vesicles Released From *Aeromonas* Strains Are Involved in the Biofilm Formation

**DOI:** 10.3389/fmicb.2020.613650

**Published:** 2021-01-07

**Authors:** Soshi Seike, Hidetomo Kobayashi, Mitsunobu Ueda, Eizo Takahashi, Keinosuke Okamoto, Hiroyasu Yamanaka

**Affiliations:** ^1^Laboratory of Molecular Microbiological Science, Faculty of Pharmaceutical Sciences, Hiroshima International University, Hiroshima, Japan; ^2^Laboratory of Medical Microbiology, Department of Health Pharmacy, Yokohama University of Pharmacy, Yokohama, Japan; ^3^Collaborative Research Center of Okayama University for Infectious Diseases in India, National Institute of Cholera and Enteric Diseases, Kolkata, India

**Keywords:** *Aeromonas*, biofilm, outer membrane vesicles (OMVs), extracellular polymeric substances (EPS), extracellular matrix (ECM)

## Abstract

*Aeromonas* spp. are Gram-negative rod-shaped bacteria ubiquitously distributed in diverse water sources. Several *Aeromonas* spp. are known as human and fish pathogens. Recently, attention has been focused on the relationship between bacterial biofilm formation and pathogenicity or drug resistance. However, there have been few reports on biofilm formation by *Aeromonas*. This study is the first to examine the *in vitro* formation and components of the biofilm of several *Aeromonas* clinical and environmental strains. A biofilm formation assay using 1% crystal violet on a polystyrene plate revealed that most *Aeromonas* strains used in this study formed biofilms but one strain did not. Analysis of the basic components contained in the biofilms formed by *Aeromonas* strains confirmed that they contained polysaccharides containing GlcNAc, extracellular nucleic acids, and proteins, as previously reported for the biofilms of other bacterial species. Among these components, we focused on several proteins fractionated by SDS-PAGE and determined their amino acid sequences. The results showed that some proteins existing in the *Aeromonas* biofilms have amino acid sequences homologous to functional proteins present in the outer membrane of Gram-negative bacteria. This result suggests that outer membrane components may affect the biofilm formation of *Aeromonas* strains. It is known that Gram-negative bacteria often release extracellular membrane vesicles from the outer membrane, so we think that the outer membrane-derived proteins found in the *Aeromonas* biofilms may be derived from such membrane vesicles. To examine this idea, we next investigated the ability of *Aeromonas* strains to form outer membrane vesicles (OMVs). Electron microscopic analysis revealed that most *Aeromonas* strains released OMVs outside the cells. Finally, we purified OMVs from several *Aeromonas* strains and examined their effect on the biofilm formation. We found that the addition of OMVs dose-dependently promoted biofilm formation, except for one strain that did not form biofilms. These results suggest that the OMVs released from the bacterial cells are closely related to the biofilm formation of *Aeromonas* strains.

## Introduction

*Aeromonas* spp. are Gram-negative, facultative anaerobic rod-shaped bacteria and are ubiquitously distributed in diverse water sources and also in foods (Janda and Brenden, 1987). To date, 36 species of *Aeromonas* have been identified (Hoel et al., 2019). Several *Aeromonas* species, such as *A. hydrophila* and *A. sobria*, are known as human pathogens that cause foodborne gastrointestinal diseases (Janda and Abbott, 2010). However, these pathogens also cause severe extraintestinal diseases in an opportunistic manner, including sepsis, peritonitis, and meningitis, especially in immunocompromised patients or elderly persons (Michael Janda and Abbott, 1998). Various kinds of extracellular virulence factors, including cytolysins, enterotoxins and proteases, are thought to be deeply involved in the pathogenesis of these conditions (Pemberton et al., 1997; Fujii et al., 1998; Okamoto et al., 2000). Other unknown factors may also be involved in the virulence of these pathogens.

In recent years, research on bacterial biofilms has been widely developed in various fields. Numerous studies have reported that bacterial biofilms attached to improperly stored medical devices can promote nosocomial infections in medical practice (Donlan, 2001; Ribeiro et al., 2012; McConoughey et al., 2014; Balan et al., 2019). With respect to *Aeromonas*, several reports have shown that some *Aeromonas* strains form biofilms on medical devices by strongly adhering to non-living surfaces such as plastic materials, leading to infectious disease in patients (Andreoli-Pinto and Graziano, 1999; Tang et al., 2014). Thus, there is a close relationship between the formation of bacterial biofilms and the onset of infectious diseases. It is therefore important to elucidate the biofilm formation process when considering the pathogenicity of bacteria.

The extracellular polymeric substances (EPS) of bacteria are generally considered to be components of the bacterial extracellular matrix (ECM) and to be fundamental to the physicochemical properties of bacterial biofilms (Costerton et al., 1999). Thus, the EPS establish the functional and structural integrity of biofilms. Sugimoto et al. (2013) have developed a simple extraction method for bacterial ECM using a high concentration of NaCl. The availability of this method has greatly facilitated the analysis of bacterial ECM components. In fact, the method has already been used in the analysis of ECM components of *Pseudomonas aeruginosa*, *Escherichia coli*, and *Propionibacterium acnes*, and the results have been reported (Chiba et al., 2015; Okuda et al., 2018).

The ECM is thought to contain various factors that are deeply involved in bacterial biofilm formation; moreover, some or all of these factors likely contribute to both the formation and structural maintenance of the ECM. Several reports have examined the roles of the EPS included in the ECM in biofilm formation (Fong and Yildiz, 2015; Limoli et al., 2015; Okshevsky et al., 2015). For example, polysaccharides, extracellular nucleic acids such as extracellular DNA molecules (eDNAs), and some matrix proteins are thought to be closely related to bacterial biofilm formation (Ma et al., 2009; Toyofuku et al., 2012). In *Vibrio cholerae*, the matrix proteins RbmA, RbmC, and Bap1, in addition to *Vibrio* polysaccharide and nucleic acids, are known as the major components of *V. cholerae* biofilm (Fong et al., 2006; Fong and Yildiz, 2007; Berk et al., 2012). Recently, the crystal structure of Bap1 has also been elucidated (Kaus et al., 2019). Thus, some bacterial biofilms are being analyzed at the molecular level to identify their components. However, information on the biofilm formed by *Aeromonas* is scarce.

In this study, therefore, we first engaged in research to clarify what factors are involved in the biofilm formation by *Aeromonas*. The results showed that the biofilms formed by several *Aeromonas* strains include polysaccharides containing *N*-acetyl glucosamine (GlcNAc), eDNAs, and proteins, as seen in other bacterial biofilms. Among these components contained in the *Aeromonas* biofilms, we found that some proteins in ECM might be closely related to the *Aeromonas* biofilm formation. The amino acid sequences of these proteins matched those of the outer membrane proteins, such as porins of Gram-negative bacteria. We further found that the outer membrane vesicles (OMVs) were released from the cells in most of the *Aeromonas* strains, and that the OMVs must be closely related to the biofilm formation in *Aeromonas* strains. We believe our present findings will provide not only the first insights toward elucidation of the molecular mechanism of *Aeromonas* biofilm formation, but also useful information for controlling the biofilm formation in *Aeromonas* infection in the future.

## Materials and Methods

### Bacterial Strains Used in This Study

The *Aeromonas* clinical and environmental strains used in this study are listed in Table 1. All experiments were approved by the university committee and were conducted appropriately in consideration of the biosafety level.

**TABLE 1:** Bacterial strains used in this study.

*Aeromonas* strain	Isolation sites and description		References
102	Clinical	*Aeromonas veronii* biotype *sobria*	Khan et al. (2008)
104	Clinical	*Aeromonas veronii* biotype *sobria*	Khan et al. (2007)
106	Clinical	*Aeromonas veronii* biotype *sobria*	Khan et al. (2008)
115	Clinical	*Aeromonas veronii* biotype *sobria*	This study
119	Clinical	*Aeromonas veronii* biotype *sobria*	Khan et al. (2008)
ATCC7966	Environmental	*Aeromonas hydrophila*	Purchased from ATCC
404	Clinical	*Aeromonas hydrophila*	This study
424	Clinical	*Aeromonas hydrophila*	This study
448	Environmental	*Aeromonas hydrophila*	This study
451	Environmental	*Aeromonas hydrophila*	Takahashi et al. (2013)
461	Environmental	*Aeromonas hydrophila*	This study
605	Clinical	*Aeromonas caviae*	This study
617	Clinical	*Aeromonas caviae*	This study

### Biofilm Formation and Measurement

*Aeromonas* strains were cultured in lysogeny broth (LB) medium at 37°C for 24 h. Overnight cultures were diluted 100-fold in LB medium in 96-well flat-bottom polystyrene plates (Corning, Glendale, AZ, United States) at 30°C for 24 h. The biofilms formed on the polystyrene plates were then washed twice with Dulbecco’s phosphate-buffered saline (DPBS; FUJIFILM Wako Pure Chemical, Osaka, Japan) and fixed with 4% paraformaldehyde in DPBS at room temperature. The biofilms were further washed twice with DPBS. After removal of the supernatants, the biofilms were stained with 1% crystal violet for 30 min and subsequently washed with DPBS. Then, 100 μL of a decolorizing agent (70% ethanol, 30% acetone) was added to the stained biofilms to extract the crystal violet dye. The level of crystal violet present in the destained solution was measured at 590 nm using an optical microtiter plate reader (Molecular Devices, Sunnyvale, CA, United States).

### Effects of DNase I and Proteinase K on the Biofilms Formed on the Polystyrene Plate

To examine the roles of the eDNAs and proteins contained in the ECMs in the structure maintenance of *Aeromonas* biofilms, *Aeromonas* strains were cultured under the same conditions as shown above except that either 100 units/mL DNase I (Roche, Basel, Switzerland) or 100 μg/mL proteinase K (Sigma-Aldrich, St. Louis, MO, United States) was added to the medium after the biofilm formation. The samples were treated with each enzyme at 37°C for 1 h.

In addition, to confirm that the DNase I and proteinase K used in the above experiments digested the eDNAs and proteins present in the ECMs, respectively, 100 units/mL DNase I or 100 μg/mL proteinase K was also added to the ECMs extracted from the bacterial cells. After treatment with each enzyme, the eDNAs and proteins were analyzed by agarose gel electrophoresis and SDS-PAGE, respectively.

### Extraction of ECMs

Extraction of the extracellular matrices (ECMs) of the bacterial cells was done according to the method reported by Chiba et al. (2015). Briefly, the procedure was carried out as follows. The bacterial cells were cultured at 30°C for 24 h in LB medium and collected by centrifugation at 8,000 rpm for 10 min at 25°C. After washing with DPBS, the cells were suspended in the extraction buffer [Tris-HCl buffer (pH 8.0) containing 1.5 M NaCl]. The extraction buffer treatment causes the release of ECMs from the bacterial cells into the buffer solution. Finally, the bacterial cells were removed by centrifugation at 8,000 rpm for 10 min at 25°C, and the supernatant solution was collected. The supernatant solution thus obtained was used as the solution containing ECMs and was stored at −30°C until use.

### Fluorescence Images

**FIGURE 1:**
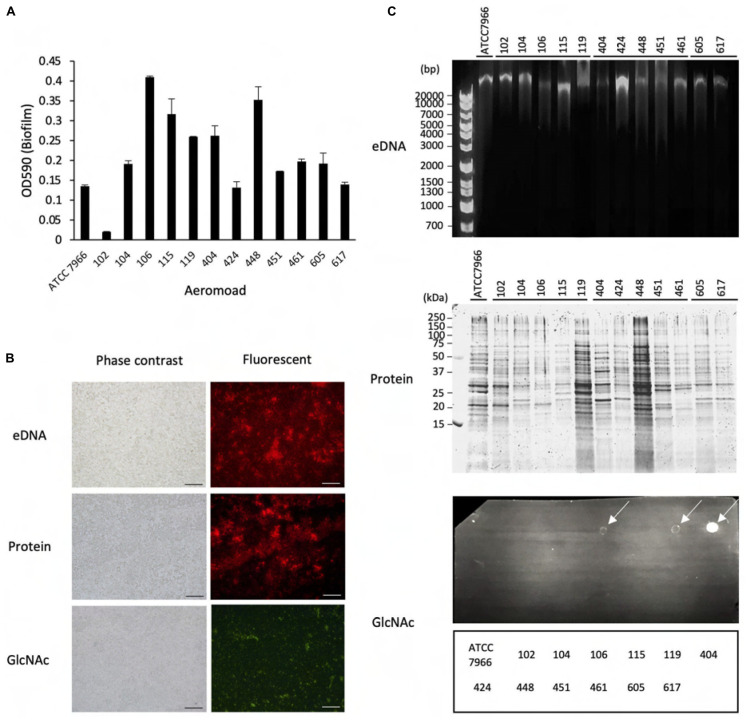
Biofilm formation by *Aeromonas* strains and their components. **(A)** Biofilm formation by *Aeromonas* strains was assessed by a microtiter plate screening assay as described in the text. Experiments were performed in triplicate on two independent occasions, and data are shown as the mean and standard deviation. **(B)** Fluorescence microscopic views of the column labeled “Fluorescent”; eDNAs, proteins and polysaccharides containing GlcNAc were detected by a fluorescence method using BOBO-3 (red), SYPRO (red) and WGA-Alexa488 (green), respectively. “TD” shows a transmitted image of cells confirmed in a bright field by fluorescence microscope. Bars represent 50 μm. **(C)** eDNAs, proteins and polysaccharides containing GlcNAc were detected by agarose gel electrophoresis, SDS-polyacrylamide gel electrophoresis and dot-blot analysis using the fluorescence reagent WGA-Alexa488. The protein bands in the SDS-polyacrylamide gel were visualized with fluorescence reagent (Oriole Fluorescent Gel Stain; Bio-Rad, Hercules, CA, United States). Detection of polysaccharides containing GlcNAc using WGA-Alexa-488 was clearly detected in *A. veronii* biotype *sobria* 106, 119 and *A. hydrophila* 404 strains (indicated by arrows), but only a few were detected in other strains.

*Aeromonas* strains were cultured in LB medium at 30°C for 24 h to form biofilm. After extraction of the ECMs, the presence of eDNAs, proteins and polysaccharides containing GlcNAc in the extracted ECMs were visualized using BOBO-3 (Thermo Fisher Scientific, MA, United States), FilmTracer SYPRO Ruby Biofilm Matrix Stain (Thermo Fisher Scientific, MA, United States) and wheat germ agglutinin (WGA)-Alexa488 (Thermo Fisher Scientific, MA, United States), respectively. BOBO-3, a cell-impermeable DNA stain reagent, was used at 250 nM to visualize eDNAs in the ECMs. FilmTracer SYPRO Ruby Biofilm Matrix Stain was treated with the sample for 60 min. After the treatment, the sample was gently washed three times with sterile water to visualize proteins in the ECMs. WGA-Alexa488 conjugate, a lectin that binds to GlcNAc, was employed at 10 μg/mL to visualize polysaccharides containing GlcNAc in the ECMs. We observed the stained samples using fluorescent microscope BX51-33-FL-2 (Olympus, Tokyo, Japan) (Figure 1) or confocal laser scanning microscope FV-300, Olympus, Tokyo, Japan) (Figures 4, 5). Each fluorescence intensity was quantified using “Image J” image analysis software (Collins, 2007; Schneider et al., 2012).

### Identification of the Proteins Existing in the Extracted ECMs

The extracted ECMs were subjected to SDS-PAGE and the gel was stained using a Silver stain MS kit (Nacalai Tesque, Kyoto, Japan). Five major protein bands detected from the electrophoretic gel were cut out, and the excised gels were sent to Japan Proteomics (Miyagi, Japan) for identification of the proteins contained in the gels. Identification of the proteins in each excised gel was carried out by nanoLC-MS/MS analysis.

### OMVs Purification

The OMVs were purified according to the methods reported by Bauman and Kuehn (2006) and Avila-Calderón et al. (2018). Briefly, *Aeromonas* strains (*A. veronii* biotype *sobria* 104 and 106 strains, also 102 strain) were cultured on LB agar plates for 24 h at 30°C. Then the bacteria were suspended in 10 mL of sterile DPBS. The bacterial suspension was centrifuged at 10,000 × *g* for 30 min. The supernatant was passed through a 0.22 μm membrane filter (NIPPON Genetics, Tokyo). Particulate components in the obtained solution were harvested by ultracentrifugation (100,000 × *g* for 2 h at 4°C) using a P65ST rotor (Hitachi, Tokyo). The pellet was washed twice with DPBS and resuspended in the sterile DPBS containing 30% (vol/vol) Optiprep (Sigma-Aldrich). The components in the suspension were further separated on Optiprep density gradients at 10% (0.9 mL), 15% (0.9 mL), 20% (0.9 mL), 25% (0.9 mL) and 30% (0.9 mL) (vol/vol). Samples were then ultracentrifuged to equilibrium (100,000 × *g* for 17 h at 4°C; P65ST rotor) and fractionated (450-μl aliquots). The protein concentration in each fractionated sample was determined using the Bradford method (Bio-Rad, Hercules, CA, United States). The presence of the OMVs in the fractionated samples was confirmed by observation with a transmission electron microscope (TEM) (see below). The sample containing purified OMVs was stored at −80°C until use.

### Transmission Electron Microscope Observation

Verification of the purified OMVs was performed by TEM observation. The OMV preparations were adsorbed onto carbon-coated copper grid (400 mesh). The samples were negatively stained with 2% (w/v) uranyl acetate. The TEM observations were carried out by the Hanaichi Electron Microscope Technical Laboratory (Aichi, Japan).

### Effect of OMV on Biofilm Formation

To determine the effect of the presence of OMVs on the formation of *Aeromonas* biofilm, the extent of biofilm formation by *Aeromonas* strains was compared in the presence or absence of the purified OMVs. *Aeromonas* strains were suspended in Hanks’ balanced salt solution (+) buffer [HBSS (+) buffer] in the presence or absence of the purified OMVs. The mixtures were incubated for 2 h at 30°C. The biofilms formed on the polystyrene microtiter plates were examined as described above. We further examined the effect of the presence of the OMVs on the formation of *Aeromonas* biofilm, after hydrolyzing the proteins localized in the OMVs by proteinase K treatment. This experiment was carried out using the OMVs obtained from *A. veronii* biotype *sobria* 104 strain. Briefly, the purified OMVs sample was treated with proteinase K (100 μg/mL) at 37°C for 2 h. Then proteinase inhibitor cocktail (PI) (Nacalai Tesque, Kyoto, Japan) was added to the sample. The sample thus obtained was used as the OMVs sample in which the proteins localized in the OMVs were hydrolyzed (PK-104 OMVs).

### Statistical Analysis

The data are the average of three samples. Error bars represent standard deviations. For multiple comparisons, one-way analysis of variance (ANOVA) was used followed by Tukey’s test (^∗^*p* < 0.05, ^∗∗^*p* < 0.01) and Dunnett’s test (^∗^*p* < 0.05, ^∗∗^*p* < 0.01) in Figures 5, 6, respectively. The statistical analysis was carried out using Easy R (Saitama Medical Center, Jichi Medical University) (Kanda, 2013).

## Results

### Microtiter Plate Assay for Assessment of the Biofilm Formation by *Aeromonas* Strains and Analysis of Constituents of the Biofilm

To examine the biofilm-formation ability of various *Aeromonas* strains, including clinical and environmental isolates, we used an *in vitro* assay system with microtiter plates (Pedersen, 1982). A total of 13 *Aeromonas* strains were tested using a microtiter plate screening assay as originally reported by O’Toole and Kolter (1998). Since the *A. hydrophila* ATCC7966 strain was already known to form a biofilm (Elhariry, 2011; Grim et al., 2013), the microtiter assay was carried out using this strain as a positive control. As shown in Figure 1A, destained biofilm (measured at 590 nm) was seen in almost all *Aeromonas* strains tested but not in the *A. veronii* biotype *sobria* 102 strain. The assay showed that both the *A. veronii* biotype *sobria* 106 and *A. hydrophila* 448 strains have more biofilm-forming potential than the other strains.

Next, we analyzed the components contained in the *Aeromonas* biofilms. In general, the bacterial ECM is already known to be the main structure supporting biofilms and to consist of eDNAs, proteins and polysaccharides (Flemming and Wingender, 2010). First, therefore, to examine whether eDNAs, proteins and polysaccharides containing GlcNAc are contained on the cell surface (including the ECM) of *Aeromonas* strains, these constituents were specifically detected using fluorescent substances. Figure 1B shows a typical fluorescence microscopic view of the eDNAs, proteins, and polysaccharides containing GlcNAc detected on the cell surface of the *A. veronii* biotype *sobria* 106 strain. This result clearly demonstrated that the cell surface of this strain contains eDNAs, proteins, and polysaccharides containing GlcNAc.

To determine whether these components exist in the bacterial ECM, we next isolated the ECM, which forms the framework of the bacterial biofilms, and analyzed its constituents. As expected, the ECM extracted from the *Aeromonas* strains also contained eDNAs, proteins and polysaccharides containing GlcNAc. Although it is likely that these components of the ECM function to sustain the biofilm formed in *Aeromonas* strains, the results showed that the amount of each of these ECM constituents varied among the bacterial strains. In particular, only a few strains in which polysaccharides containing GlcNAc were markedly detected (Figure 1C, indicated by arrows).

### Effect of Addition of DNase and Proteinase K to the *Aeromonas* Biofilms

The results in Figure 1 revealed that the ECM, which is thought to play an important role in maintaining the bacterial biofilm, contains eDNAs, proteins and polysaccharides containing GlcNAc in various *Aeromonas* strains. In this experiment, it was found that eDNAs and proteins were detected in the ECMs of almost all *Aeromonas* strains, but the number of strains having an ECM containing a large amount of polysaccharides including GlcNAc, was limited (Figure 1C). We therefore consider that eDNAs and proteins, if not other ECM components as well, appear to play an indispensable role in maintaining the biofilm constructed by *Aeromonas* strains. To examine this possibility, we next examined how the presence of protease or DNase affects the biofilm formed by *Aeromonas* strains. For this purpose, we used proteinase K and DNase I.

**FIGURE 2:**
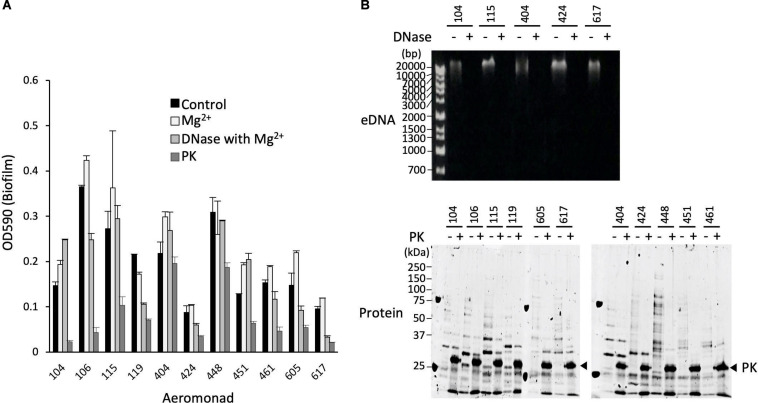
Effect of proteinase K and DNase I on structure maintenance of the biofilms formed by *Aeromonas*. **(A)** After forming the biofilms of various *Aeromonas* strains, either proteinase K (100 μg/mL) or DNase I (100 units/mL) was added to the medium to examine the disintegration of the biofilms. Since Mg^2+^ is required for the expression of DNase I activity, the effect of adding Mg^2+^ alone was also examined as a control experiment in the absence of DNase I. **(B)** Degradation of eDNAs and proteins in the ECMs by the addition of DNase I (DNase+) and proteinase K (PK+), respectively, was verified by agarose gel electrophoresis (upper panel) and SDS-polyacrylamide gel electrophoresis (lower panel), respectively. The arrowhead indicates proteinase K (PK). The protein bands in the SDS-polyacrylamide gel were visualized with fluorescence reagent (Oriole Fluorescent Gel Stain; Bio-Rad, Hercules, CA, United States).

As shown in Figure 2A, addition of DNase I (100 units/mL) with Mg^2+^ did not significantly degrade the biofilm formed by any of the *Aeromonas* strains tested, although the eDNAs existing in the ECMs of several *Aeromonas* strains were completely digested by the DNase I activity (Figure 2B, upper panel). In contrast, the amounts of biofilms formed by almost all *Aeromonas* strains except for the *A*. *hydrophila* 404 strain were markedly decreased by addition of proteinase K (100 μg/mL) (Figure 2A). The proteins contained in the ECMs were also digested by proteinase K (Figure 2B, lower panel). In addition, we confirmed that neither DNase I alone nor proteinase K alone had any effect on the survival of the bacterium, because the result of counting of the viable bacterial cells was almost the same even in a culture with either DNase I (100 unit/mL) or proteinase K (100 μg/mL) (data not shown). These results indicate that the proteins contained in the ECMs are largely responsible for the maintenance of the *Aeromonas* biofilm structure.

### Identification of the Proteins Present in the *Aeromonas* ECMs

**FIGURE 3:**
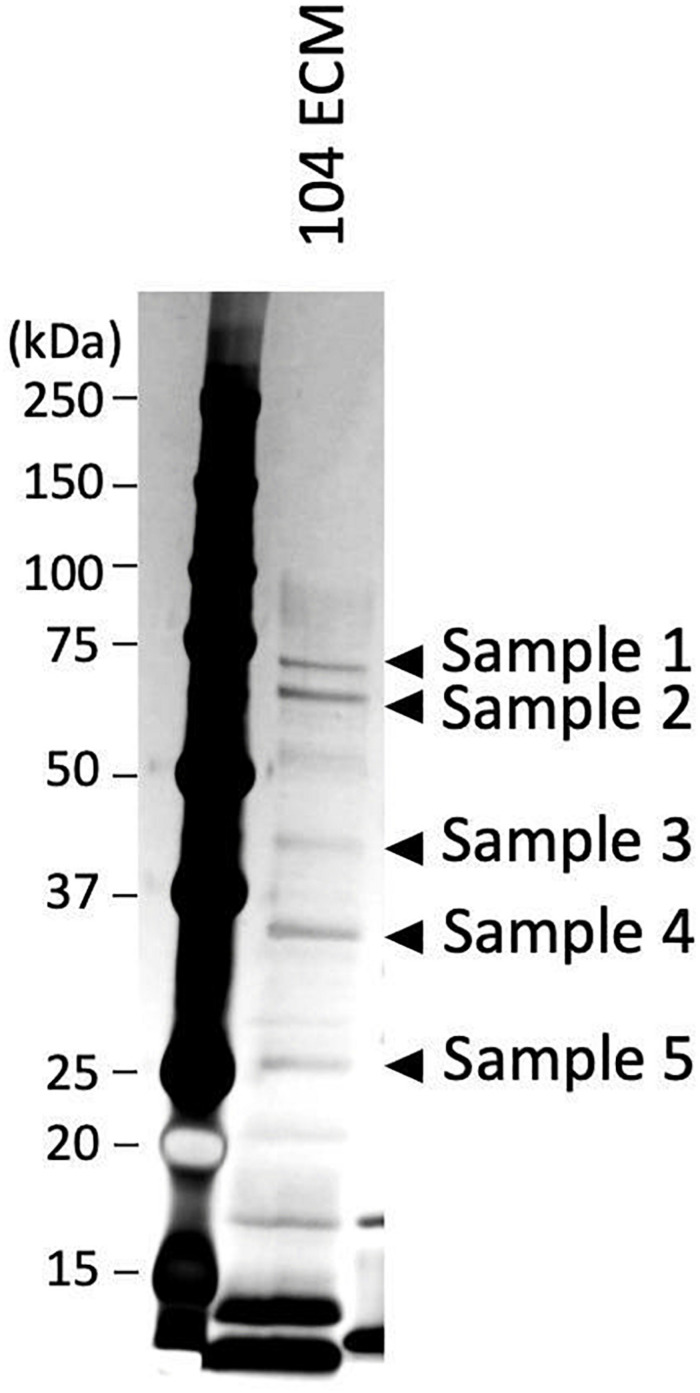
Fractionation of the proteins present in the ECMs of *A*. *veronii* subtype *sobria* 104 strain by SDS-polyacrylamide gel electrophoresis. After silver staining, the protein bands of samples 1 to 5 shown in the figure were cut out to extract the proteins, and the proteins extracted were analyzed by a nano-LCMS/MS system.

To identify the proteins that contribute to the maintenance of the *Aeromonas* biofilm structure, we identified the proteins existing in the ECMs using a nano-LCMS/MS system. Although the proteolytic fragments produced by proteinase K treatment were diverse, it seems that the samples obtained from the *A. veronii* biotype *sobria* 104 strain have more bands showing the same molecular weight as the proteolytic fragments present in the samples obtained from other *Aeromonas* strains (Figure 2B, lower panel). Furthermore, it also seems that the bands of proteolytic fragments were clearly separated in the sample derived from the 104 strain. We therefore used the ECMs extracted from the *A. veronii* biotype *sobria* 104 strain for protein analysis using the nano-LCMS/MS. Five major protein bands were cut out from the gel fractionated by SDS-PAGE (Figure 3) and the obtained samples were applied to the nano-LCMS/MS system.

The data showed several structural protein candidates derived from each protein sample, as shown in Table 2. These candidate proteins included the structural proteins localized on the outer membrane of Gram-negative bacteria such as vitamin B12 transporter BtuB, maltoporin, porins, and OmpA. We therefore speculate that the proteins derived from the outer membrane of *Aeromonas* strains may be closely related to sustaining the biofilm structure.

**TABLE 2:** Identification of the proteins present in the *Aeromonas* ECMs.

Sample		Protein	MW	Score	Accession
Sample 1	1	Lipase [*Aeromonas* sp. ASNIH3]	82,288	517	*WP_042864705.1*
	2	Nuclease [*Aeromonas* sp. ASNIH3]	76,581	408	*WP_103259634.1*
Sample 2	1	Hypothetical protein [*Aeromonas caviae*]	66,930	981	*WP_049637039.1*
	2	Hypothetical protein [*Aeromonas rivipollensis*]	66,906	244	*WP_106885298.1*
	3	Vitamin B12 transporter BtuB [*Aeromonas* sp. L_1B5_3]	66,920	69	*WP_043851718.1*
Sample 3	1	Maltoporin [*Aeromonas caviae*]	45,122	588	*KEP92336.1*
Sample 4	1	Porin [*Aeromonas rivipollensis*]	38,091	154	*WP_106886400.1*
	2	Outer membrane porin II (OmpK40) [*Aeromonas salmonicida* (strain A449)]	38,455	121	*WP_005320112.1*
Sample 5	1	Porin OmpA [*Aeromonas* sp. ASNIH3]	36,013	554	*WP_010673501.1*

It has already been established that Gram-negative bacteria actively release OMVs under certain conditions (Beveridge, 1999). In the *Pseudomonas aeruginosa* PAO1 strain, it was reported that OMVs were present within the matrix formed by the bacterial biofilm (Schooling and Beveridge, 2006; Toyofuku et al., 2012). It is therefore likely that OMVs or the proteins derived from OMVs contribute to maintenance of the biofilm structure. Accordingly, we further investigated whether the OMVs contribute to the biofilm formation of *Aeromonas*.

### The Role of OMVs in the Bacterial Biofilm Formation by *Aeromonas* Strains

**FIGURE 4:**
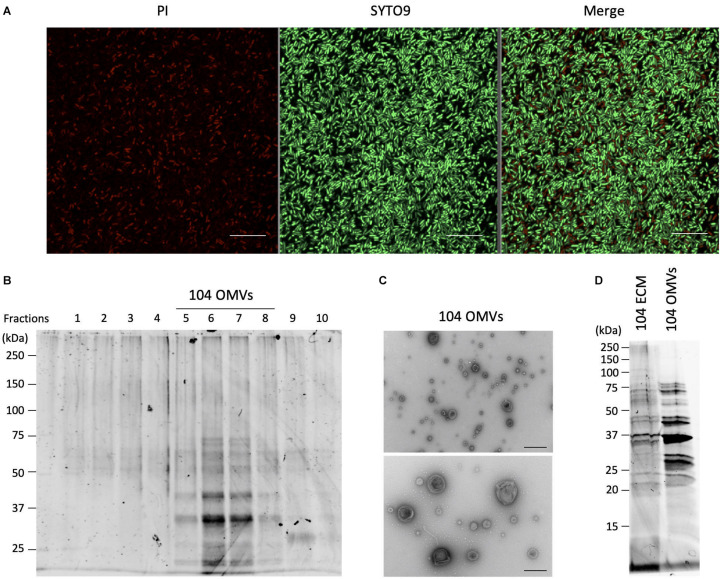
Isolation and purification of OMVs from the ECMs fraction. **(A)** Live and dead bacterial cells were fluorescently detected with SYTO9 and propidium iodide (PI), respectively. Bars represent 10 μm. **(B)** Isolation and purification of the OMVs were performed by ultracentrifugation using the Optiprep density gradients method. Fractions 5 to 8 shown in the panel were collected as the OMVs fraction. **(C)** Purification of the OMVs was confirmed by electron microscopic observation. Bars in the upper and lower panels represent 400 nm and 200 nm, respectively. **(D)** The protein fractionation patterns of the ECMs and the purified OMVs fractions were compared by SDS-polyacrylamide gel electrophoresis. All figures representatively showed the results obtained from the experiment using *A*. *veronii* subtype *sobria* 104 strain.

To confirm that the OMVs released from the bacterial cells are involved in the biofilm formation, we first isolated and purified the OMVs from the *Aeromonas* strain. For this purpose, we used *A. veronii* biotype *sobria* 104 and 106 strains that actively form biofilms. In addition, we also used *A. veronii* biotype *sobria* 102 strain because it was observed that the 102 strain released the OMVs even though the strain did not form biofilm (Supplementary Figure 1). The purification of the OMVs was verified by observation using a TEM. As the OMVs could be similarly isolated and purified from any of *Aeromonas* strains by the isolation and purification methods described in the text, the results obtained using *A. veronii* biotype *sobria* 104 strain are shown in Figure 4 as a representative.

Since the OMVs are thought to be actively released from living bacteria to the extracellular space, we investigated how many living bacteria exist in the biofilms formed by *Aeromonas*. We used SYTO9 and propidium iodide (PI) to fluorescently detect the live and dead bacterial cells, respectively. SYTO9 is a membrane-permeable DNA-staining reagent that stains live cells significantly, while PI is a membrane non-permeable reagent which thus stains only the DNA of cells with damaged cell membranes (dead cells). As shown in Figure 4A, observation of the *Aeromonas* cells after formation of the biofilm revealed that most of the cells in the biofilm were alive, although there were also some dead bacteria. Under this condition, we next isolated and purified the OMVs from the biofilm formed by *Aeromonas*.

The isolation and purification of the OMVs were done by ultracentrifugation using the Optiprep density gradients method. SDS-PAGE analysis of each fraction obtained by the ultracentrifugation showed that the fractions with 20–25% (vol/vol) Optiprep density contained the OMVs (Figure 4B). To verify the presence of the OMVs in these fractions, electron microscopic observation using TEM was performed (Fernandez-Moreira et al., 2006; Avila-Calderón et al., 2018). As expected, these fractions clearly contained the OMVs (Figure 4C). As seen in Figure 4A, the biofilm contained mostly living cells, such that the purified OMVs were likely to have been secreted from living cells.

We further examined the protein profile of the OMVs to compare it with that of the ECM. The results of the SDS-PAGE analysis showed that the electrophoretic pattern of the proteins contained in the OMVs was very similar to that in the ECM (Figure 4D), suggesting that a large amount of the OMVs were included in the ECM constituting the bacterial biofilm. We therefore speculated that the OMVs may be closely related to formation of the *Aeromonas* biofilm formation.

**FIGURE 5:**
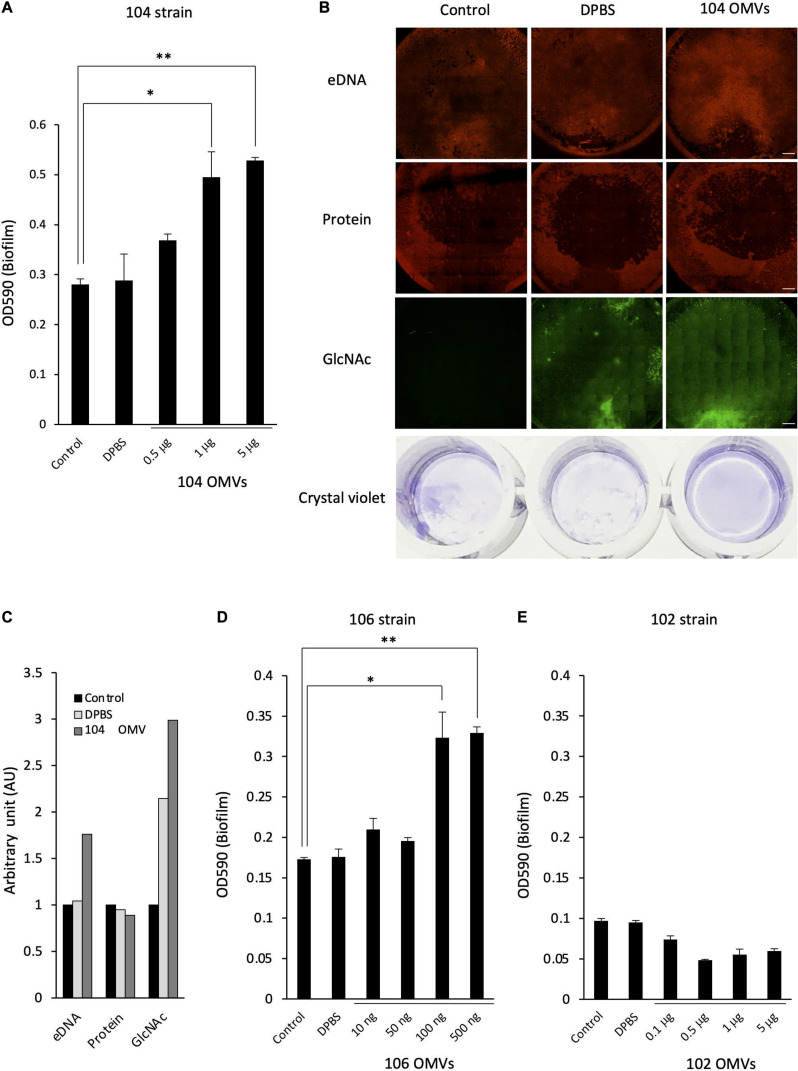
Effect of the OMVs on the biofilm formation by *Aeromonas*. The degree of the biofilm formation was examined under various coexisting amounts of the OMVs purified from the *A*. *veronii* subtype *sobria* strains, 104 strain **(A)**, 106 strain **(D)**, and 102 strain **(E)**. Addition of Dulbecco’s phosphate-buffered saline (DPBS) alone did not increase the biofilm formation, confirming that DPBS did not affect the biofilm formation by *Aeromonas* (bar graph labeled DPBS). One-way ANOVA was used followed by Tukey’s test to assess significance. Values are the mean ± standard deviations. *p < 0.05; **p < 0.01. **(B)** The increase in the amount of the biofilm formed by the addition of the purified OMVs was also confirmed by fluorescently detecting the amounts of eDNAs (by using BOBO-3), proteins (by using SYPRO), and polysaccharides containing GlcNAc (by using WGA-Alexa488). Bars represent 500 μm. The figures showed the results of the experiment using *A*. *veronii* subtype *sobria* 104 strain. **(C)** The graph shows the results of quantifying the fluorescence intensities of BOBO-3, SYPRO, and WGA-Alexa488 shown in Figure 5B. The fluorescence intensity was quantified using Image J software.

To verify our hypothesis, we next examined the effect of the presence of the OMVs on the formation of the bacterial biofilm by *Aeromonas*. When the *A. veronii* biotype *sobria* 104 strain was cultured in the presence of the purified OMVs obtained from the 104 strain (104 OMVs), we found that the biofilm-forming ability of the strain was further enhanced depending on the amount of 104 OMVs added (Figure 5A). When we performed an experiment similar to that shown in Figure 1B, the eDNAs, proteins and GlcNAc in polysaccharides contained in the ECM forming the bacterial biofilm were more prominently observed when the *Aeromonas* strain was cultured with the purified OMVs (Figure 5B, rightmost panels). The increase in both eDNAs and GlcNAc-containing polysaccharides was also indicated by image analysis using Image J analysis software (Figure 5C). Similarly, when the *A. veronii* biotype *sobria* 106 strain having remarkable biofilm-forming ability was cultured with the purified OMVs obtained from the 106 strain (106 OMVs), the promotion of biofilm formation by the addition of 106 OMVs was also observed as in the case of the 104 strain (Figure 5D). These results suggest that the OMVs are closely involved in the biofilm formation of this *Aeromonas* strain. In addition, the promotion of biofilm formation in the 106 strain was caused to the same extent as that in the 104 strain with the addition of a smaller (about 10 times less) amount of 106 OMVs (Figure 5D).

In contrast, when the *A. veronii* biotype *sobria* 102 strain with very low biofilm-forming ability was cultivated with the purified OMVs derived from the 102 strain, no promotion of biofilm formation was observed with the addition of the OMVs (Figure 5E). We think that this result is a very interesting finding. That is, this result means that the presence of the OMVs may not simply promote the formation of the bacterial biofilms, but that the bacteria which will receive the OMVs must also have certain conditions to cause the promotion of biofilm formation.

### Involvement of the Proteins Localized on the OMVs in the Biofilm Formation by the *Aeromonas* Strain

The above study revealed that the OMVs increase the biofilm formation by *Aeromonas*. In addition, the results shown in Figure 2 indicated that the proteins present in the ECMs contribute to the maintenance of the *Aeromonas* biofilm structure. From these results, we speculate that the proteins localized on the OMVs may affect the biofilm formation by *Aeromonas*. To explore this possibility, we further examined the effect of proteolytic digestion of the proteins present in the OMVs in the biofilm formation. We performed this experiment using purified OMVs from the *A. veronii* biotype *sobria* 104 strain.

**FIGURE 6:**
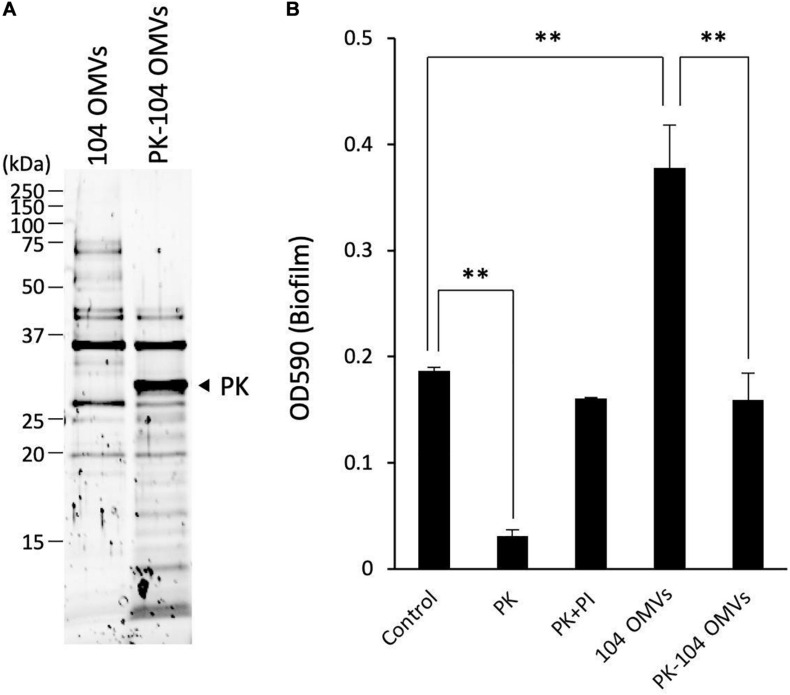
Involvement of the proteins localized on the OMVs in the biofilm formation by *Aeromonas*. **(A)** The purified OMVs were pretreated with proteinase K (PK). Degradation of the proteins localized on the OMVs was confirmed by SDS-polyacrylamide gel electrophoresis. **(B)** The increase in the biofilm formation caused by the addition of the purified OMVs was attenuated by pretreatment of the OMVs with proteinase K (PK-104 OMVs). One-way ANOVA was used followed by Dunnett’s test to assess significance. Values are the mean ± standard deviations. ^∗∗^*p* < 0.01.

As shown in Figure 6A, several proteins contained in the OMVs were hydrolyzed by the treatment with proteinase K. After treatment of the OMVs with proteinase K, an inhibitor cocktail solution for proteinase K was added to the reaction mixture in order to stop the proteolytic action of proteinase K. The sample mixture thus treated was designated as PK-104 OMVs.

As already shown in the results of Figure 2, addition of proteinase K caused a decrease of the biofilm formation by the *A. veronii* biotype *sobria* 104 strain (Figure 6B, PK). This decrease in the biofilm formation was inhibited by addition of the proteinase K inhibitor cocktail (Figure 6B, PI). On the other hand, the biofilm formation by the *A. veronii* biotype *sobria* 104 strain was further increased by addition of the OMVs purified from the same strain, as already shown in the results of Figure 5 (Figure 6B, 104 OMVs).

To examine whether the proteins present in the OMVs affect the increase in biofilm formation caused by the addition of the OMVs, we observed the biofilm formation by the *A. veronii* biotype *sobria* 104 strain in the presence of PK-104 OMVs. The results clearly demonstrated that, unlike the addition of purified OMVs, the addition of the PK-104 OMVs did not induce an increase in the biofilm formation (Figure 6B, PK-104 OMVs). This finding indicates that some proteins present in the OMVs may be involved in the formation of the *Aeromonas* biofilm.

## Discussion

It is widely known that biofilm formation contributes not only to the viability and pathogenicity of bacteria in infected hosts, but also to the ability of bacteria to survive in poor environments such as those depleted of nutrient sources (Tolker-Nielsen, 2015). Further, there are many cases in which nosocomial infections are caused as a result of bacteria contaminating medical devices and forming biofilms (Donlan, 2001; Ribeiro et al., 2012; McConoughey et al., 2014; Balan et al., 2019). Thus, it is considered that the formation of bacterial biofilms is deeply involved in the adaptation to environmental changes and the exertion of pathogenicity.

Several reports have been reported that *Aeromonas* strains also form biofilms, and that such biofilm formation is associated with adaptation to various environmental stresses and pathogenicity (Nagar et al., 2017; Talagrand-Reboul et al., 2017). Biofilm formation has also been shown to contribute to the multidrug resistance of *Aeromonas* (Thenmozhi et al., 2014; Dias et al., 2018). However, little is known about the molecular mechanism by which the biofilm formation of *Aeromonas* proceeds.

In this study, we analyzed the molecular components involved in the bacterial biofilm formation by *Aeromonas* strains and investigated factors that promote the biofilm formation. First, we investigated the biofilm-forming ability using thirteen *Aeromonas* clinical and environmental strains and an *in vitro* plate assay system. We found that all the *Aeromonas* strains but one (*Aeromonas veronii* biotype *sobria* 102 strain) had the ability to form biofilms (Figure 1A). Studies using the *Aeromonas veronii* biotype *sobria* 106 strain have shown that the ECM components of the biofilm formed by this strain were composed of eDNAs, proteins and polysaccharides containing GlcNAc (Figure 1B), meaning that the constituents of the biofilm formed by *Aeromonas* strains are likely similar to those of the biofilm formed by other bacteria such as *Pseudomonas* strains (Talagrand-Reboul et al., 2017). The biofilm components of the other *Aeromonas* strains having biofilm-forming ability were similar, but it was considered that the content of polysaccharides containing GlcNAc differed among the strains examined (Figure 1C). We therefore consider that the eDNAs and proteins existing in ECMs may play an important role in maintaining the *Aeromonas* biofilm.

Analysis using proteinase K and DNase I clearly showed that the degradation of proteins significantly affected the maintenance of the *Aeromonas* biofilms, whereas the degradation of eDNAs did not (Figure 2). This result means that some protein contained in the ECMs plays an indispensable role in maintaining the structure of the *Aeromonas* biofilms. It has been reported that *Staphylococcus aureus* expresses the protein determinants engaging in the biofilm formation and that Esp, a serine protease secreted by commensal *S*. *epidermidis*, disassembles preformed biofilms of *S. aureus* and inhibits its colonization (Sugimoto et al., 2013). Thus, a certain, as-yet-unidentified protein must be closely related to the maintenance of *Staphylococcal* biofilms. A recent report revealed that both matrix Bap 1 and Rbm C proteins produced by *Vibrio cholerae* play distinct adhesive roles in its biofilm formation (Kaus et al., 2019). Wrobel et al. (2020) have recently reported that two autotransporters of *Yersinia ruckeri*, which localize to the outer membrane, contribute to biofilm formation. It is therefore likely that several proteins present in the ECMs also make major contributions to the biofilm formation in *Aeromonas* strains.

To identify the proteins that are involved in the biofilm formation by *Aeromonas* strains, we fractionated the proteins contained in the ECMs by SDS-polyacrylamide gel electrophoresis. In carrying out this experiment, we used the ECMs obtained from the *A*. *veronii* biotype *sobria* 104 strain because the bands of those fragments were clearly separated in the sample from the *A. veronii* biotype *sobria* 104 strain (Figure 2B, lower panel). After performing SDS-polyacrylamide gel electrophoresis, the protein bands shown in Figure 3 (samples 1 to 5) were cut out, and the proteins were analyzed by nano-LCMS/MS. We found that most of the proteins contained in the samples were thought to be outer membrane proteins (Table 2). We therefore suspect that the ECMs may contain large amounts of protein components derived from the outer membrane proteins.

It has been shown that Gram-negative bacteria actively release outer membrane-derived membrane vesicles (OMVs) to outside the cells (Beveridge, 1999). With respect to the possible function of OMV production in Gram-negative bacteria, there are generally considered to be three possibilities. One is that the OMV production functions to mediate the extracellular secretion of various pathogenic factors (Kadurugamuwa and Beveridge, 1995, 1996; Wai et al., 2003; Kesty and Kuehn, 2004; Kesty et al., 2004; Kuehn and Kesty, 2005; Mashburn and Whiteley, 2005). The second possibility is that OMVs may function in the transport of quorum-sensing inducers that contribute to bacterial signal transduction (Mashburn and Whiteley, 2005; Tashiro et al., 2010; Inaba et al., 2015). The third possibility is that OMVs contribute to the formation of biofilms, as they have been shown to do in studies of *Pseudomonas aeruginosa* (Schooling and Beveridge, 2006; Schooling et al., 2009; Cooke et al., 2019). As mentioned above, we found that several proteins in the ECMs that are considered to constitute *Aeromonas* biofilms were derived from the outer membrane. We therefore considered that OMVs might be involved even in the formation of *Aeromonas* biofilms.

To examine this possibility, we purified the OMVs from the *A*. *veronii* biotype *sobria* 104 and 106 strains that having remarkable biofilm-forming ability and examined how the purified OMVs affected *Aeromonas* biofilm formation. In addition, we also purified the OMVs from the 102 strain and did a similar experiment because we found that the *A. veronii* biotype *sobria* 102 strain released the OMVs even though the strain did not form biofilm (Supplementary Figure 1). The OMVs from the *Aeromonas* strains were thought to be properly purified by the method using Optiprep density gradients ultracentrifugation.

Then, we compared the extent of the biofilm formation of each *Aeromonas* strain in the presence of the purified OMVs obtained from the same strain. In the *A*. *veronii* biotype *sobria* 104 and 106 strains that having remarkable biofilm-forming ability, we found that the amount of the biofilm formed by both *Aeromonas* strains increased as the added amount of purified OMVs increased (Figures 5A,D). Fluorescence image analysis using *A*. *veronii* biotype *sobria* 104 strain also revealed that, in particular, the both amounts of eDNAs and GlcNAc in polysaccharides which are considered to constitute biofilms increased as the added amount of purified OMVs increased (Figure 5B). We therefore propose that the increase of extracellular OMVs promotes the biofilm formation by the *Aeromonas* strain. Similar findings have already been reported in studies of *P*. *aeruginosa* biofilm (Schooling and Beveridge, 2006; Cooke et al., 2019), *Helicobacter pylori* (Yonezawa et al., 2009), *Vibrio cholerae* (Fong et al., 2006; Fong and Yildiz, 2007; Berk et al., 2012), and *V. fischeri* (Shibata and Visick, 2012). Among them, the study using the *H. pylori* TK1402 strain revealed that the biofilm formation of this strain was markedly increased following the addition of the OMV-fraction in a dose-dependent manner. These findings strongly support our proposal. Thus, our study suggests that, at least in the case of the *A. veronii* biotype *sobria* 104 and 106 strains, the biofilm formation is markedly promoted by the presence of the OMVs produced by the strain itself, as previously observed for the *H. pylori* TK1402 strain (Yonezawa et al., 2009).

In addition, the promotion of biofilm formation in the 106 strain was caused to the same extent as that in the 104 strain with the addition of a smaller (about 10 times less) amount of 106 OMVs as shown in Figure 5D. This result means that the 106 strain is more receptive to the effects of 106 OMVs. If the *Aeromonas* strains have a receptor for the OMVs, the 106 strain may express more receptors for the OMVs than the 104 strain. Alternatively, if biofilm formation is promoted by the intracellular signal transduction system after the bacteria have reacted with the OMVs, such system of the 106 strain may operate more sensitively than that of the 104 strain after receiving the action of the OMVs. We further proceed with detailed studies at the molecular level and clarify these points in future.

Interestingly, however, when the *A. veronii* biotype *sobria* 102 strain with very low biofilm-forming ability was cultured with the purified OMVs obtained from the 102 strain, no promotion of biofilm formation was observed with the addition of the OMVs (Figure 5E). We think that this result has very important implications. This is because, despite the presence of the OMVs, the promotion of biofilm formation is unlikely to occur unless the bacteria have certain properties. As mention in the previous paragraph, it is likely that *Aeromonas* strains may have receptors for the OMVs and/or a unique signal transduction system after receiving the action of the OMVs. We assume the *A. veronii* biotype *sobria* 102 strain may lack such receptor for the OMVs and/or may be deficient in such signal transduction system, so that the promotion of biofilm formation did not occur even in the presence of the OMVs.

We further investigated which factors in OMVs are responsible for promoting biofilm formation of the *Aeromonas* strain. The results in Figure 2 showed that addition of proteinase K caused a decrease in the biofilm formation by *Aeromonas* strains, indicating that some protein in the ECMs may be closely related to the formation of biofilm. We predicted that a certain protein localized on the OMVs might be involved in *Aeromonas* biofilm formation.

To investigate this possibility, we examined the biofilm formation of the *Aeromonas* strain in the presence of the OMVs treated with proteinase K. As shown in Figure 6, the enhancement of biofilm formation by the OMVs was attenuated when the OMVs were pre-treated with proteinase K. This result indicated that a certain protein localized on the OMVs might contribute to the biofilm formation by the *Aeromonas* strain, although it is not possible to discuss what proteins may engage in this function at present. We speculate that such proteins localized on the OMVs may act on the surface receptor of the *Aeromonas* strain to promote biofilm formation. We also speculate that such proteins on the OMVs may not function to the *A. veronii* biotype *sobria* 102 strain because of lacking the surface receptor. At this point of view, we would like to further investigate the molecular mechanism of the action of the OMVs on *Aeromonas* strain in more detail and report the results again in future.

Earlier studies revealed that polysaccharides are major matrix components related to the biofilm formation, while subsequent studies showed that proteins, lipids and nucleic acids are also important contributors to biofilm formation (Flemming and Wingender, 2001; Branda et al., 2005). Schooling et al. (2009) reported that the interactions of DNA with the OMVs influenced OMV surface properties and affected the reactivity of the interactions of matrix polymers and other constituents that are thought to be important for biofilm formation. Thus, it is considered that changes in the surface structures of OMVs greatly affect the interaction between bacterial surface layers. Therefore, we also speculate that the surface layer structure of OMVs may change when the proteins on the surface of OMVs are hydrolyzed by the action of proteinase K, and such change may diminish the promotion of biofilm formation by OMVs. To investigate this possibility, we would like to proceed with further analysis at a molecular level.

Several papers have already reported that *Aeromonas* strains form biofilms and adapt to various environmental changes, including antibiotic resistance (Elhariry, 2011; Nagar et al., 2017; Talagrand-Reboul et al., 2017; Dias et al., 2018). To date, however, few papers have discussed the molecular mechanism of biofilm formation. In the present study, we showed that the OMVs released outside the cells enhance biofilm formation in the *Aeromonas* strains having biofilm-forming ability. We believe that this will be an important finding for exploring the molecular mechanism of the biofilm formation by *Aeromonas*. Further, it is likely that a certain protein existing on the OMVs may be involved in the biofilm formation by *Aeromonas*. Du et al. (2016) have reported that the oligopeptidase PepF is negatively related to the biofilm formation by *A*. *hydrophila*. This finding may be related to our present finding that the degradation of the OMV proteins by the action of proteinase K reduces *Aeromonas* biofilm formation.

Since our preliminary data showed that other *Aeromonas* strains also produce and release OMVs into the extracellular space and almost all strains form the biofilms, it is conceivable that the OMVs widely contribute to the biofilm formation in other *Aeromonas* strains having biofilm-forming ability. However, it is still too early to conclude that the OMVs uniformly promote biofilm formation in any *Aeromonas* strains, because the OMVs formed by the other strains may not be uniform in their shapes and properties. It is therefore necessary to consider that some factors other than the OMVs may also influence *Aeromonas* biofilm formation. From this perspective, we must proceed with a more detailed study of the molecular mechanism of biofilm formation in *Aeromonas* strains.

## Data Availability Statement

The datasets presented in this study can be found in online repositories. The names of the repository/repositories and accession number(s) can be found in the article/Supplementary Material.

## Author Contributions

SS and HY designed the study, supervised the experiments, and wrote the manuscript. SS performed the experiments and analyses. HK performed the experiments and supervision. MU contributed to data curation. ET and KO contributed to collection of the *Aeromonas* samples. All authors contributed to the article and approved the submitted version.

## Conflict of Interest

The authors declare that the research was conducted in the absence of any commercial or financial relationships that could be construed as a potential conflict of interest.

## Supplementary Material

Supplementary Figure 1 Isolation and purification of OMVs from the ECMs fraction obtained from *A*. *veronii* subtype *sobria* 102 strain. Although the *A*. *veronii* subtype *sobria* 102 strain did not show biofilm-forming ability, the strain markedly released the OMVs. Therefore, we also isolated and purified the OMVs from the strain using the same technique as shown in Figure 4. **(A)** Isolation and purification of the OMVs were performed by ultracentrifugation using the Optiprep density gradients method. Fractions 6 to 9 shown in the panel were collected as the OMVs fraction. **(B)** Purification of the OMVs was confirmed by electron microscopic observation. Bars in the upper and lower panels represent 400 nm and 200 nm, respectively.
